# Involvement, leadership and social practice to the development of postgraduate attributes: evidence from extracurricular education with Chinese characteristics

**DOI:** 10.3389/fpsyg.2023.1161308

**Published:** 2023-05-30

**Authors:** Sen Li, Yusheng Hu, Lingling Wang, Enjun Xia

**Affiliations:** ^1^School of Management and Economics, Beijing Institute of Technology, Beijing, China; ^2^School of Information Management, Beijing Information Science and Technology University, Beijing, China; ^3^School of Economics and Management, China University of Petroleum, Qingdao, China

**Keywords:** postgraduate attribute, extracurricular education, student involvement, student leadership, social practice

## Abstract

Limited by students’ time and energy, participation in extracurricular activities is not necessarily beneficial to the development of postgraduate attributes. Thus, it is necessary to explore the impact path of extracurricular activities and education outcomes on the development of postgraduate attributes. From a configuration perspective, this study identifies the asymmetric causal effects of engagement and extracurricular education on postgraduate attributes. First, this study proposes a theoretical framework for postgraduate attribute development in extracurricular education with Chinese characteristics based on the input-environment-output (IEO) theory. Second, 166 academic scholarship applications submitted by the whole third-grade postgraduates who are from a science and engineering school at a double first-class university in China are taken as the sample. Finally, utilizing data envelopment analysis (DEA) and fuzzy set qualitative comparative analysis (fsQCA), this study conducts the effect of the combination of causal conditions on the development of postgraduate attributes. Results are as follows: (1) the development efficiency of postgraduate attribute in extracurricular education with Chinese characteristics is practical but still insufficient; (2) four configurations consistently linked to high development efficiency of postgraduate attributes. Specifically, in context with outstanding academic research achievement and excellent moral character, participating in extracurricular education or not consistently linked to high development efficiency. In contrast, in a context characterized by academic achievement or moral award not outstanding enough, involvement in extracurricular activities or social practice is consistently linked to high development efficiency. In addition, no configuration links student leadership to high development efficiency, and non-scientific research ability is consistently linked to low development efficiency; (3) there is an asymmetric causal relationship between the high and low development efficiency paths, indicating that the conditions affecting the development of postgraduate attributes have multiple concurrencies. These findings provide a new practical path and perspective for promoting the development of postgraduate attributes through extracurricular education with Chinese characteristics.

## 1. Introduction

Student development is some kind of positive change that occurs in the student, such as intellectual and ethical growth, cognitive complexity, self-awareness, and social identity (Patton et al., 2016). Rodgers (1990) defined student development as the ways that a student grows, progresses, or increases his or her developmental capabilities as a result of enrollment in an institution of higher education. Higher education serves several fundamental roles in that it imparts knowledge, develops students’ professional skills, promotes other academic and professional outcomes, and shapes students’ attributes and qualities (Anderson et al., 2013). Archer and Davison (2008) found that personal attributes, such as communication skills, integrity, self-confidence, and personality, were among the top 10 qualities that employers thought important when recruiting graduates. These professional skills and personal attributes must be acquired through formal and informal experiences in different learning spaces (Jackson, 2008), with academic study and extracurricular activities (ECAs) representing other spaces within the student experience (Barnett, 2011).

Numerous studies have reported that extracurricular activity participation (ECAP) during adolescence is positively associated with academic performance (Bartko and Eccles, 2003; Roeser and Peck, 2003; Peck et al., 2008; Guilmette et al., 2019), psychological health (Viau and Poulin, 2015), persistence (Lufi and Tenenbaum, 1991; Allen et al., 2015), self-regulation (Hansen et al., 2003), and social interaction (Mahoney et al., 2005). Similarly, research on the value of ECAs in higher education has shown that ECAP benefits academic achievement (Terenzini et al., 1996; Shulruf, 2010; Van der Zanden et al., 2018) and that the quality of ECAP significantly predicts adaptation and transition to university (Harvey et al., 2006; Tieu and Pancer, 2009; Tieu et al., 2010). For example, data drawn from the U.S. National Survey of Student Engagement indicate that spending more time engaging in extracurricular activities increases students’ likelihood of acquiring valuable work skills and attributes such as improved critical thinking, leadership, and social skills (Kuh, 1995; Tieu et al., 2010; Thompson et al., 2013). In university settings, ECAP could also promote civic engagement, prosocial activities, and social integration (Fredricks and Eccles, 2006) as well as decreased internalization problems such as symptoms of depression and anxiety (Bartko and Eccles, 2003).

Extracurricular education with Chinese characteristics has become an essential supplement to classroom learning since 1983 (Zhu et al., 1983). Many Chinese scholars have demonstrated that extracurricular education with Chinese characteristics has unique functions and advantages with regard to cultivating students’ comprehensive quality (Song and Zeng, 2018; Chen et al., 2019; Xiao and Ren, 2021), and this field has gradually developed into a new front for the comprehensive implementation of quality-oriented education in China. For example, the Central Committee of the Communist Youth League and the Ministry of Education jointly proposed the university extracurricular activity record system in 2016 (CYD, 2018). This record system covers innovation and entrepreneurship, social practice, culture and art, sports activities, work history, skills and expertise, ideological growth (Chen et al., 2019). At the end of 2017, the Central Committee of the Communist Youth League started to pilot and promote this system on a larger scale (CYD, 2018). By May 2018, 507 universities and 291 junior colleges in China had implemented the university extracurricular activity record system, and 427 universities and 234 junior colleges had issued or were on the verge of releasing relevant documents (CYD, 2018). Generally, all colleges and universities in China have reached a preliminary consensus regarding the implementation of the university extracurricular activity record system (CYD, 2018).

It is worth noting that, although these domestic and foreign findings have highlighted the association between ECAP and educational outcomes, not all such studies have reported a positive association in this context (Lindsay and Paton-Saltzberg, 1994). Moreover, due to the limitations of research methods, more evidence supporting causal effects is needed (Shulruf, 2010). In addition, given the richness of extracurricular education with Chinese characteristics and the limited time and energy of students, increasing numbers of participants in extracurricular education entail a reduction in academic investment. The mention fact leads to the following reflections: “—Is extracurricular education better for students? —How can students achieve effective development through extracurricular education?” Student development is like a systematic project that is affected not only by input factors but also by output factors which, in turn, affect the further development of students (Zou and Tan, 2013). The impacts of various factors on student development are not independent, and the factors have different combination effects through linkage and matching (Zepke et al., 2011). Therefore, it is necessary to explore how extracurricular education affects the development of students from a configuration perspective.

Based on student development theory and graduate attribute theory, this study proposes a theoretical framework for postgraduate attribute development in extracurricular education with Chinese characteristics. Based on a combination of DEA and fsQCA, this study aims to identify the multiple concurrent causalities (Ragin, 2009a) that might improve/hinder postgraduate attribute development. The sample consists of 166 academic scholarship applications submitted by the whole third-grade postgraduates who are from a science and engineering school at a double first-class university in China. The primary contributions of this study can be summarized as follows: (1) based on the IEO model, this study develops a collaborative linkage framework of postgraduate attribute development in extracurricular education with Chinese characteristic; (2) using the DEA method, this study measures the efficiency of the development of postgraduate attributes and verifies the effectiveness of extracurricular education with Chinese characteristics; and (3) from the configurational perspective, this study proposes four practical ways of promoting postgraduate attribute development in extracurricular education with Chinese characteristics, which are refined into an output-driven model and an input-output linkage model.

## 2. Theoretical background

### 2.1. Student development theory

According to different perspectives, student development theories can be divided into psychosocial, cognitive-structural, typological, and person-environment schools (Patton et al., 2016). Psychosocial theories focus on analyzing the content of student development and emphasize the critical issues that students face in college. Representative theories include Erikson’s life cycle theory (Erikson, 1994) and Chickering’s seven-vector development theory (Chickering and Reisser, 1993). Cognitive-structural theories focus on describing the process of students’ epistemological and intellectual development that occurs during college (Kohlberg, 1969; Perry, 1999). Typological theories treat differences among students as relatively fixed characteristics reflected in the cognitive process. Kolb (2007) and Myers and Myers (2010) are representative authors. Person-environment theories regard student development as the result of the interaction between individuals and the environment, such as Astin’s (1970) IEO model and student involvement theory (Astin, 1984), Pascarella’s comprehensive causality model (Pascarella, 1985), and Tinto’s student departure theory (Tinto, 2012).

Astin’s IEO model is one of the most frequently used frameworks for understanding the effects of college on a range of outcomes of undergraduate education (Gayles et al., 2012), which has been used extensively in higher education settings for nearly four decades (Stein, 2007). The model includes three major components—inputs, environments, and outputs. Inputs refer to the personal traits, attitudes, and characteristics of students themselves, such as gender, race, high school grade point average, expectations, and involvement. Environments include the programs, strategies, interventions, peers’ influences, and policies introduced to the student while they are in college. Outcomes are the consequences and/or results that are derived through the influence of the input and environment variables, such as academic achievement, critical thinking disposition, and social-emotional adjustment (Van der Zanden et al., 2018).

### 2.2. Graduate attributes

Graduate attributes are the qualities, skills, and understandings that a university community agrees that its students should develop during their time at the institution (Bowden et al., 2000). These attributes include the disciplinary expertise or technical knowledge that has traditionally formed the core of most university courses, and the qualities that prepare graduates to become agents of social good in an unknown future (Bowden et al., 2000). Many studies have claimed that these attributes are core outcomes of higher education and have thus emphasized the importance of graduate attributes with regard to employability and social-civic responsibility. Barrie (2007) identified four distinct orientations: precursory, complementary, translation and enabling. In the most complex integrated conception, graduate attributes are understood to be interwoven aptitudes and abilities, such as academic inquiry and intellectual curiosity, the ability to accommodate diversity and alternative perspectives, the ability to create and defend ideas, and the ability to employ communication as a vehicle for learning (Barrie, 2007). While these outcomes might lie at the heart of formal academic and university curricula, the processes by which students develop these abilities may also extend far beyond the regular academic classroom. In other words, graduate attributes develop through student engagement during the learning experience that occurs due to the individual’s immersion in the university’s intellectual and social community, of which formal courses are only one part. Moreover, student-centered approaches to the development of graduate attributes are consistently associated with better graduate attribute outcomes. Therefore, universities’ efforts to develop effective student-centered strategies serve to guarantee the quality of university teaching (Prosser and Barrie, 2003).

### 2.3. Research framework

Drawing on the research conducted by Bowden et al. (2000), this study defines postgraduate attributes in terms of the qualities, skills, and understandings that a university community agrees that its students should develop during their time at the institution. It is worth noting that the postgraduate attributes referenced in this study are based on the most complex integrated conception summarized by Barrie (2007). These attributes include not only disciplinary expertise or technical knowledge, which lie at the core of the university curriculum, but also communication ability, social ability, student leadership, a sense of responsibility, and the ability to serve society. Consequently, this study follows Barrier’s emphasis on student participation in the broader university experience and the critical importance of designing student-centered teaching strategies.

**FIGURE 1:**
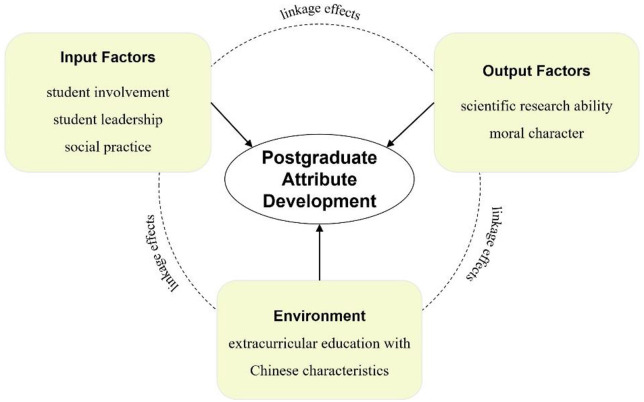
Research framework of postgraduate attribute development in extracurricular education with Chinese characteristic.

Therefore, based on Astin’s IEO model, this study regards extracurricular education with Chinese Characteristics as a dimension of the university environment, and divides the effect of extracurricular education on postgraduate attribute development into three parts: the student involvement, student leadership role, and social practice of prosocial behavior in the extracurricular education as *input factors*; the extracurricular education with Chinese characteristics as *environment*, scientific research ability and moral character as *output factors* corresponding to academic education and moral education in China. Notably, postgraduate attribute development is a systematic project with the linkage effects among input factors, environment, and output factors (Zou and Tan, 2013). The research framework is drawn in Figure 1.

### 2.4. Research hypotheses

#### 2.4.1. Student involvement and postgraduate attribute development

Student involvement is now considered to be an overarching meta-construct in which an ecosystem of students, educators, service staff, and institutions interact to create enriching tertiary experiences (Zepke, 2014; Kahu and Nelson, 2018). Bowden et al. (2021) defined student involvement as a student’s positive social, cognitive, emotional, and behavioral investments in the context of interacting with their tertiary institution and its focal agents, such as peers and employees. These four dimensions jointly motivate student striving, persistence, and retention within academic contexts (Kuh et al., 2008).

For example, involvement has strong links with the emotional dimension of engagement, as the more involved a student is, the higher that student’s propensity for the creation of intrinsic value, such as joy, elation, enjoyment, passion, pride (Kahu, 2013; Pansari and Kumar, 2017). Involvement can also motivate the social dimension of engagement, such as by enhancing feelings of connection, belonging, warmth, and relatedness between students and their peers, staff, and the institution community (O’Keeffe, 2013). In addition, positive involvement is central to academic achievement (De Carolis et al., 2020). Thus, we propose:

Hypothesis 1: Postgraduates who are more involved in extracurricular education with Chinese characteristics are more likely to get a higher development efficiency of the postgraduate attribute.

#### 2.4.2. Social practice and postgraduate attribute development

Many scholars have called for more authentic forms of instruction based on Dewey’s notion of experiential learning (Dewey, 1986) and Bandura’s social learning theory (Bandura and Walters, 1977). The social practice represents a potentially powerful form of pedagogy because all types of social practice activities involve the incorporation of academic learning with practical service (Salam et al., 2019). Various scholars have noted that social practice offers a variety of benefits for students, such as a deep understanding of course contents (Dienhart et al., 2016), increased student practical experience (Meyer et al., 2016), acquiring specific career-oriented skills (Geller et al., 2016), enhancing social responsibility and civic leadership (Weiler et al., 2013). Therefore, we propose:

Hypothesis 2: Postgraduates who participate more in social practice are more likely to get a higher development efficiency of the postgraduate attribute.

#### 2.4.3. Student leadership and postgraduate attribute development

Scholars have agreed that one of the core functions of universities is to prepare the leaders of tomorrow’s society (Astin and Astin, 2000). Developing leadership in students is increasingly becoming an explicit priority of higher education institutions (Skalicky et al., 2020). This is because universities face the challenge of graduate employability, which has prompted institutions to pay attention to the development of graduate attributes, including leadership (Osmani et al., 2015). Student leadership development within the higher education context comes in a variety of forms, including those that are specifically designed to develop leadership capabilities within a program (e.g., student union, leadership award programs), and those that provide leadership experience incidentally through student-facing activities (e.g., tutoring peers, campus tour guides) or community volunteer activities (Skalicky et al., 2020). All students who involve themselves in leadership education have the potential to increase their skills and knowledge (Zimmerman-Oster and Burkhardt, 1999; Komives et al., 2006; Wren, 2013). Therefore, we propose:

Hypothesis 3: Postgraduates who take on a student leadership role are more likely to get a higher development efficiency of the postgraduate attribute.

#### 2.4.4. Scientific research ability and postgraduate attribute development

Scientific research ability is an essential indicator of the quality of graduate education (Zajda and Rust, 2016; Van der Zanden et al., 2019). From the social perspective, postgraduates’ scientific research ability directly affects national scientific and technological innovation and comprehensive competitiveness. Therefore, the British government identified the following as the primary goal of postgraduate education in 1974,which is meeting the future talent needs of the country, providing further training for qualified, appropriate, and keen students; and contributing to the advancement of knowledge (Clark and Clark, 1993).

The National Science Foundation of the United States implemented a strategic framework for postgraduate education investment from 2016 to 2020 (Huang et al., 2021). China’s Ministry of Education also implemented a postgraduate education innovation plan in 2003–2007 to improve postgraduate innovation ability. Cultivating scientific research ability is a continuous and uninterrupted process, which is not only related to curriculum system design, academic environment, tutor relationships, but also to extracurricular social practice, community culture, and psychological capital (Zhou and Wang, 2018). Therefore, we propose:

Hypothesis 4: Postgraduates with a higher scientific research ability are more likely to get a higher development efficiency of the postgraduate attribute.

#### 2.4.5. Moral character and postgraduate attribute development

Moral education practice has become a student-centered practice, and incorporated into various fields, such as music, health, and business (Julia et al., 2020). Recently, moral character has developed into an essential and popular theme in various countries (Rissanen et al., 2018; Darnell et al., 2019). For example, increasing numbers of schools in the U.S. have incorporated character education into their programs (Narvaez and Lapsley, 2008). A comprehensive civics and moral education program was established in Singapore, which focuses on nurturing both moral and character development and active citizenship and aims to equip students with appropriate competencies and value systems (Koh, 2012). In China, moral education belongs to citizenship education (Zhao and Tan, 2007). Integrative character education is not a package of virtues designed to control student behavior, but rather a continuation of moral development through role building, interactions among students, families, and the community, as well as student active participation, discussion and reflection (Marshall et al., 2011). The research conducted by Marshall et al. (2011) shows that comprehensive character education can improve students’ prosocial and moral behaviors and their reading and test scores. Therefore, we propose:

Hypothesis 5: Postgraduate students with a higher moral character are more likely to get a higher development efficiency of the postgraduate attribute.

## 3. Methodology

### 3.1. Research method

Fuzzy set qualitative comparative analysis is a variant of qualitative comparative analysis that combines fuzzy sets with logical principles in the context of qualitative comparative analysis (Ragin, 2009b). Compared with other variance-based quantitative techniques, fsQCA aims to disentangle the relationship of these combinations of variables to a given outcome (Fiss, 2007) by expressing the relative contribution of a set of variables to a given outcome rather than individual contributions (Vis, 2012). The essential features of this approach are as follows: (1) fsQCA is based on Boolean algebra and its associated tools, such as truth tables, consistency, coverage scores, and set coincidences (Ragin, 2014). (2) fsQCA is a method of data analysis used for small and medium sample cases that combines the case orientation of qualitative methods and the variable orientation of quantitative methods, thereby combining case analysis and cross-case comparison. However, larger samples can also be investigated (Vis, 2012). (3) fsQCA is based on combinations of variables, including equilibria, that is, different paths leading to specific outcomes (Fernández-García et al., 2020). This approach is used to explore the generative logic across different conditional configurations and outcomes and to identify the synergistic effects of multiple conditional variables based on an acknowledgment of the complexity of causality (Du and Jia, 2017).

The development of postgraduate attributes is a systematic project in which input and output factors interact with each other, and the impacts of each factor are not independent. These impacts have different combined effects through linkage matching. Therefore, to explore the multiple driving mechanisms underlying the promotion of postgraduate attribute development in extracurricular education with Chinese characteristics, this study uses fsQCA to examine the multiple conjunctural causations between student input and output in the context of a university environment featuring extracurricular education.

### 3.2. Sample and data collection

Referring to the study by Guilmette et al. (2019), this study selected a science and engineering school at a double first-class university in China. Given that the output of postgraduates will increase gradually over time, this study took a random sample consists of academic scholarship applications which are submitted by the whole third-grade postgraduates voluntarily. Data collection was carried out in September 2022. Finally, 166 valid academic scholarship applications were obtained. Notably, the extensive coverage of graduate academic scholarships for as many as 90% of students guarantees the diversity of applicants and research value. The proportions of special prizes, first prizes, second prizes, and non-awarded applicants in the sample are 22.30, 21.69, 42.78, and 13.25%, respectively, thus ensuring maximum heterogeneity in the sample and avoiding excessive consistency within the sample.

### 3.3. Variable measurement

Conditional variables in this study include student involvement, social practice, student leadership, academic achievement, and moral development, all measured by objective indicators included in the scholarship applications, all of which are fuzzy values. The measurements of these variables are displayed in Table 1.

**TABLE 1:** Variable list.

Construct	Measurement	References
Student involvement	The number of participants in extracurricular activities (e.g., science and technology activities, competitions, cultural and sports activities, and other activities)	Khademi Ashkzari et al., 2018; Skalicky et al., 2020
Social practice	The total score of volunteer service, summer social practice, and winter social practice	Scales et al., 2006
Student leadership	The work performance score of the student leadership role	Skalicky et al., 2020
Scientific research ability	The academic achievement score of published papers and authorized patents	Thursby, 2000; Bonaccorsi et al., 2006; Abramo et al., 2008
Moral character	The honor and award scores in the scholarship applications	Kou, 2011
Postgraduate attribute development	The total efficiency is calculated through the input-oriented CRS model of DEA, in which student involvement, social practice, and student leadership are input variables, and scientific research ability, moral development are output variables	Amara et al., 2020

#### 3.3.1. Student involvement (SI)

The behavioral dimension is the most frequently measured dimension within national barometers of the student experience (Kuh et al., 2008; Zepke, 2014). According to the research by Khademi Ashkzari et al. (2018), this study selects the number of participants in extracurricular activities (e.g., science and technology activities, competitions, cultural and sports activities, and other activities) as the measurement of student involvement in extracurricular education with Chinese characteristics.

#### 3.3.2. Social practice (SP)

Referring to the research by Scales et al. (2006), this study selects the total score of volunteer service, summer social practice, and winter social practice in the scholarship applications as the measurement of students’ social practice in extracurricular education with Chinese characteristics.

#### 3.3.3. Student leadership (SL)

According to the research by Skalicky et al. (2020), the work performance score of the student leadership role in the scholarship applications is selected as the measurement of student leadership.

#### 3.3.4. Scientific research ability (SR)

According to the studies by Thursby (2000), Bonaccorsi et al. (2006), Abramo et al. (2008), this study selects the academic achievement score in the scholarship applications, that is, the scores of published papers and authorized patents, as the measurement of scientific research ability.

#### 3.3.5. Moral character (MC)

A moral sense of honor is the praise and award people get after fulfilling their moral obligations. This study refers to the research of Kou (2011) and selects the honor and award scores in the scholarship applications as the measurement of students’ moral character.

The outcome variable of this study is *Postgraduate Attribute Development* (PAD). Referring to the research of Amara et al. (2020), total efficiency is calculated through the input-oriented CRS model of DEA, in which student involvement, social practice, and student leadership are input variables, and scientific research ability, moral development are output variables. The formula for calculating PAD is shown in formula (1):


(1) P⁢A⁢D=t⁢o⁢t⁢a⁢l⁢e⁢f⁢f⁢i⁢c⁢i⁢e⁢n⁢c⁢y×(S⁢I+S⁢P+S⁢L+S⁢R+M⁢C)

## 4. Results

### 4.1. Efficiency assessment

This study developed an input-oriented CRS efficiency model featuring two outputs (scientific research ability and moral character) and three inputs (student involvement, social practice, and student leadership). DEAP-XP1 software was used to obtain the DEA efficiency scores for the development of postgraduate attributes in extracurricular education with Chinese characteristics.

As shown in Table 2, in extracurricular education with Chinese characteristics, the average efficiency score of the sample is 62.15%, and approximately half of the postgraduates exhibited strong DEA efficiency (development efficiency = 1). However, more than 40% of postgraduates did not achieve 50% efficiency, and nearly 45% of postgraduates did not achieve 75% efficiency. These findings show that extracurricular education with Chinese characteristics can be substantially improved with regard to its ability to promote development efficiency with regard to postgraduate attributes.

**TABLE 2:** Development efficiency of postgraduates in extracurricular education with Chinese characteristics.

Development efficiency (%)	Number	Proportions (%)
<25	44	26.5
25–49.99	23	13.9
50–74.99	7	4.2
75–99.99	8	4.8
100	84	50.6
Total	166	100

### 4.2. Variable calibration

The first step in fsQCA is a process known as calibration, in which context each case is assigned a degree of membership in each set (Ragin, 2009b). Three thresholds (full membership, a crossover point of maximum membership ambiguity, and full non-membership) are required to rescale interval variables into fuzzy sets. The research conducted by Ragin (2009b), Fiss (2011) indicated that theoretical and empirical knowledge should be taken into full consideration when selecting these three thresholds. The full membership point can be set at the 10th percentile, the 20th percentile, or the upper percentile; the crossover point can be set at the average or the median; and the full non-membership point can be set at the 80th percentile or the 90th percentile (Sun and Li, 2021).

In this study, considering the distribution characteristics of the case data (Table 3), we use sample-dependent anchors to define set membership (Gupta et al., 2020). Specifically, the three thresholds of student involvement, moral character, and postgraduate attributes development are set as follows: the 90th percentile is the anchor for full membership; the 10th percentile is the anchor for non-membership; and the median is the crossover point anchor. Similarly, the three thresholds of scientific research ability, social practice, and student leadership are set as follows: the 90th percentile is the anchor for full membership; the 10th percentile is the anchor for non-membership; and the mean is the crossover point anchor. The calibration values of the outcomes and conditions are shown in Table 4. This study uses these calibration values and fsQCA 3.1 software to calibrate the variable data.

**TABLE 3:** Descriptive statistics and percentile analysis of the outcomes and conditions.

	SI	SP	SL	SR	MC	PAD
SD	5.962	1.996	1.321	4.052	2.044	8.392
Max	33.000	10.000	6.300	19.000	10.000	41.002
Min	0.001	0.001	0.001	0.001	0.001	0.000
M	5.389	1.120	0.445	2.129	1.039	6.819
P_5	0.001	0.001	0.001	0.001	0.001	0.002
P_10	0.001	0.001	0.001	0.001	0.001	0.004
P_20	1.000	0.001	0.001	0.001	0.001	0.204
P_25	1.000	0.001	0.001	0.001	0.001	0.539
P_30	2.000	0.001	0.001	0.001	0.001	1.004
P_50 (Md)	4.000	0.001	0.001	0.001	0.100	3.579
P_70	5.800	1.000	0.001	1.000	0.500	8.096
P_75	6.000	1.000	0.001	2.000	1.181	10.416
P_80	8.000	2.000	0.001	3.330	2.000	12.402
P_90	13.700	4.000	1.000	8.500	3.650	21.288
P_95	19.000	6.000	4.000	10.875	5.100	25.376

SD, standard deviation; Max, maximum; Min, minimum; M, mean; Md, median; P_5, 5th percentile; P_10, 10th percentile; P_20, 20th percentile; P_30, 30th percentile; P_50, 50th percentile; P_70, 70th percentile; P_75, 75th percentile; P_80, 80th percentile; P_90, 90th percentile; P_95, 95th percentile.

**TABLE 4:** Calibration values of the outcomes and conditions.

	Thresholds		
	**Full membership point**	**Crossover point**	**Full non-membership** **point**
SI	0.001 (10th percentile)	4.000 (Median)	13.700 (90th percentile)
SP	0.001 (10th percentile)	1.120 (Mean)	4.000 (90th percentile)
SL	0.001 (10th percentile)	0.445 (Mean)	1.000 (90th percentile)
SR	0.001 (10th percentile)	2.129 (Mean)	8.500 (90th percentile)
MC	0.001 (10th percentile)	0.100 (Median)	3.650 (90th percentile)
PAD	0.001 (10th percentile)	3.579 (Median)	21.288 (90th percentile)

### 4.3. Necessity analyses

Before conducting a sufficiency analysis, necessity analyses must be conducted to determine whether any condition is necessary for obtaining high or low PAD. A condition is necessary if it must be present for the outcome to occur (Ragin, 2009b). Consistency thresholds for necessity analyses are typically set above 0.90 (Ragin, 2009a,b; Schneider and Wagemann, 2012; Afonso et al., 2018; Grau and López, 2018), and a condition may be considered to be necessary if it has high consistency and coverage scores. The necessity analysis results concerning high PAD show that the consistency of each condition is lower than 0.9 (Table 5), indicating that no condition is necessary for the occurrence of high postgraduate attribute development. That is, the explanatory power of any single condition on high PAD is insufficient. Therefore, the effect of a combination of conditions on high PAD must be analyzed in further detail. Correspondingly, the necessity analysis for low PAD shows that the consistency of weak scientific research ability (∼SR) is higher than 0.9, thus suggesting that weak scientific research ability may be necessary to explain low postgraduate attribute development. However, the consistency of the other conditions are all lower than 0.9, thus indicating that further analysis of the causal condition combinations impacting low PAD is needed.

**TABLE 5:** Necessary conditions for high and low development efficiency of postgraduate attributes.

	High PAD		Low PAD	
	**Consistency**	**Coverage**	**Consistency**	**Coverage**
SI	0.684965	0.673975	0.475727	0.596689
∼SI	0.590112	0.468933	0.740067	0.749657
SP	0.498192	0.720549	0.297085	0.547725
∼SP	0.687294	0.434086	0.848426	0.683064
SL	0.256331	0.575286	0.228027	0.652355
∼SL	0.845100	0.462018	0.851544	0.593435
SR	0.530805	0.855176	0.230951	0.474303
∼SR	0.673701	0.407311	0.929481	0.716331
MC	0.680580	0.741992	0.379751	0.527758
∼MC	0.566844	0.417569	0.814349	0.764698

### 4.4. Sufficiency analysis

According to fsQCA truth-table approach, this study constructs and analyzes a data matrix known as the truth table using fsQCA 3.1, which includes 32 rows (2^5^ = 32; the number of explanatory conditions is five). Each row represents a logically possible combination of conditions. Using Boolean algebra, the truth table is reduced into a simplified expression of combinations linked to the outcome. To simplify the truth table, we follow the criteria suggested by Gupta et al. (2020): (1) a frequency threshold for the minimum number of cases that must belong to a combination for that combination to be considered by the analysis and (b) a consistency benchmark to identify combinations that are reliably linked to an outcome. Therefore, this study sets the case frequency threshold at the minimum critical value of one and chooses a raw consistency threshold of 0.75 (Rihoux and Ragin, 2008), which is supplemented by a 0.7 proportional reduction in consistency (PRI) threshold (Greckhamer et al., 2018). In addition, in the section on counterfactual analysis, this study does not presuppose the direction, and all factors are measured in terms of existence or absence. Namely, the development of postgraduate attributes is a system of interactions among input, environment, and output, which is challenging to judge in a uniform manner. This study uses the Quine-McCluskey method to obtain intermediate, parsimonious, and complex solutions (Mustafa et al., 2022). Furthermore, the intermediate solution is the main solution, which is supplemented by the parsimonious solution and used to identify the core and peripheral conditions (Fiss, 2011).

To understand the results more accurately, we follow the convention of using a configuration chart to report a combination of parsimonious and intermediate solutions, as shown in Table 6. Core conditions are contained in the intermediate and parsimonious solutions, while peripheral conditions are contained in the intermediate solution but not is the parsimonious solution (Pappas and Woodside, 2021). In Table 6, black circles represent the presence of a causal condition, whereas crossed circles indicate the absence of such a causal condition. Large circles represent core conditions, whereas small circles indicate peripheral conditions. Blank spaces indicate a state of “do not care.”

**TABLE 6:** Sufficient conditions for the development efficiency of postgraduate attributes.

	High PAD				Low PAD			
	**1**	**2**	**3**	**4**	**1**	**2**	**3**	**4**
SI	–	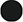	–	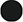	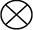	–	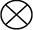	–
SP	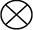	–	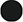	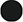	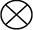	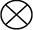	–	–
SL	–	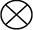	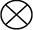	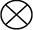	–	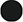	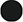	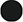
SR	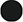	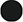	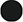	–	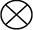	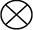	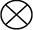	
MC	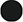	–	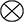	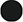	–	–	–	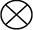
Consistency	0.989	0.983	1.000	0.905	0.958	0.965	0.870	0.943
Raw coverage	0.280	0.277	0.141	0.268	0.632	0.138	0.142	0.158
Unique coverage	0.100	0.066	0.013	0.123	0.517	0.009	0.008	0.023
Overall solution consistency	0.936				0.938			
Overall solution coverage	0.517				0.704			

Black circles represent the presence of a causal condition, whereas crossed circles indicate the absence of such a causal condition. Large circles represent core conditions, whereas small circles indicate peripheral conditions. Blank spaces indicate a state of “do not care.”

Table 6 shows that four configurations of student involvement, leadership, social practice, scientific research ability, and moral character, are consistently linked to high PAD. These configurations can be refined into the output-driven model (Configuration H_1_) and the input-output linkage model (Configurations H_2_, H_3_, and H_4_). The overall consistency and coverage are 0.936 (>0.80) and 0.517 (>0.10), respectively, which are higher than the thresholds suggested by Ragin (2009a,b), Woodside (2014), indicating that these four configurations have good explanatory power and are sufficient to ensure high development efficiency with regard to postgraduate attributes. The overall solution coverage is 0.517, which indicates that the four configurations can explain 51.7% of the empirical cases. Moreover, the raw coverage of the four configurations ranges between 0.141 and 0.280, which is higher than 0.10 and can thus be considered satisfactory (Eng and Woodside, 2012). Similarly, Table 6 shows four configurations linked to low PAD, the overall consistency and coverage values of which are 0.938 (>0.80) and 0.704 (>0.10), respectively. Moreover, the raw coverage of the four sufficient conditions ranges between 0.138 and 0.632, indicating that these conditions serve as a compelling explanation for low development efficiency with regard to postgraduate attributes.

### 4.5. Robustness test

To ensure the accuracy of the results, we conducted a stability test. First, the consistency threshold was increased from 0.75 to 0.80. Second, the PRI consistency was increased from 0.70 to 0.75. The results were all consistent with the configuration solutions shown in Table 6. Therefore, the configuration results obtained in this study are robust (Greckhamer et al., 2018; Yuan et al., 2022).

## 5. Discussion

### 5.1. Configuration path of high PAD

Configuration H_1_ (SR*MC*∼SP) shows that the configuration of scientific research ability, moral character, and a lack of social practice is linked to high development efficiency with regard to postgraduate attributes. The consistency is 0.989 (>0.800), and the raw coverage is 0.280, indicating that this path can explain approximately 28.0% of cases (Leppänen et al., 2019). The number of example cases representing this configuration is 19. Accordingly, compared with other conditions, strengthening scientific research ability and moral traits is particularly important with respect to improving the development efficiency of postgraduate attributes.

Configuration H_1_ is consistent with studies concerning policy and research on higher education effects, which have generally used scientific research ability as an essential indicator of student success, academic achievement, or the quality of graduate education (Zajda and Rust, 2016; Van der Zanden et al., 2019). This configuration is also consistent with the emphasis on scientific research achievements in international/national accreditation (De Boer et al., 2015). Furthermore, Configuration H_1_ validates the claim that postgraduate attributes are a multidisciplinary concept that includes various outcomes (Wegner, 2008; Universities UK, 2015). Higher education should not only focus on imparting knowledge (Mayhew et al., 2016) but should also include moral education and character formation. Moral character is both the starting point and a critical point for education (Wolf-Wendel and Ruel, 1999; Podger et al., 2010). Therefore, higher education should focus on cultivating students’ essential qualities and behavioral standards, shaping students’ moral responsibilities (Shaaban, 2005; Podger et al., 2010), and providing students with appropriate abilities and value systems (Nucci, 1997; Corrigan et al., 2007; Marshall et al., 2011). This approach is consistent with the central role of moral character in improving the development efficiency of postgraduate attributes emphasized in Configuration H_1_.

Configuration H_2_ (SR*SI*∼SL) shows that the configuration of scientific research ability, student involvement, and a lack of student leadership is linked to high development efficiency with regard to postgraduate attributes. The consistency is 0.983 (>0.800), and the raw coverage is 0.277, indicating that this path is able to explain approximately 27.7% of cases. With the exception of conducting scientific research, Configuration H_2_ highlights participation in extracurricular education such as scientific and technological activities, cultural and sports activities, and competitions. This path is consistent with some findings regarding the value of extracurricular activities. For example, extracurricular involvement helps enhance individual adaptive self-regulation (Larson and Verma, 1999; Hansen et al., 2003; Tieu and Pancer, 2009) and learning persistence (Allen et al., 2015), which are helpful for scientific research (Nonis et al., 2006). Participation in extracurricular activities can also induce positive feelings, such as a sense of connection with others, and allow the participant to obtain many valuable job skills or attributes, such as improved critical thinking, leadership, and social skills (Tieu et al., 2010). However, more participation in extracurricular education is not guaranteed to be beneficial for postgraduate attribute development due to the student’s limitations with regard to time and energy. Previous studies have revealed that extracurricular involvement hinders academic research (Lindsay and Paton-Saltzberg, 1994) and that this negative effect depends on individual characteristics and circumstances. Therefore, the task of achieving a good balance between academic research and extracurricular activities is challenging (Thompson et al., 2013).

Configuration H_3_ (SR*∼MC*SP*∼SL) shows that the configuration of scientific research ability, social practice, and a lack of student leadership are core conditions and that complementary non-moral character is linked to high development efficiency with regard to postgraduate attributes. The consistency is 1.000 (>0.800), and the raw coverage is 0.141, indicating that this path can explain approximately 14.1% of cases. Configuration H_3_ emphasizes the fact that while conducting scientific research, graduate students should actively participate in social practice activities associated with prosocial behaviors, such as volunteer service and community service, the cultivation of moral responsibility, and the improvement of their development efficiency and efficiency. Our findings are consistent with those of numerous studies that have identified service learning as an effective tool that can benefit students (Strage, 2000). For example, participation in community service and service learning effectively contribute to higher grades (Billig, 2004; Scales and Roehlkepartain, 2004) and academic advantages (Benson et al., 1999; Scales et al., 2006). Service learning also has a powerful impact on students’ moral, social-cognitive, and emotional development (Batchelder and Root, 1994; Ostrow, 1995; Eyler and Giles, 1996, Eyler and Giles, 1999; Kendrick, 1996; Rhoads, 1997). Participation in service learning develops students’ sense of responsibility, acceptance of diversity, and leadership skills in community work (Markus et al., 1993; Eyler and Giles, 1996; Shumer and Belbas, 1996; Brandell and Hinck, 1997; Myers-Lipton, 1998). This situation may be due to the fact that community service and service learning provide opportunities for students to experience meaningful engagement in various life settings (Hanson et al., 2003).

Configuration H_4_ (MC*SI*SP*∼SL) shows that the configuration of student involvement, social practice, moral character, and a lack of student leadership are core conditions that are linked to high development efficiency with regard to postgraduate attributes. The consistency is 0.905 (>0.800), and the raw coverage is 0.268, indicating that this path can explain approximately 26.8% of cases. Configuration H_4_ indicates that when the scientific research achievements are insufficiently outstanding, postgraduate attributes can be improved by increasing involvement in extracurricular activities and social practices associated with prosocial behaviors. For example, some students actively participate in extracurricular activities because they do not expect to make strong academic research achievements, even though extracurricular education is not a substitute for exemplary achievement. Nevertheless, extracurricular education encourages the acquisition of specific skills and personal development, which can boost employability (Thompson et al., 2013).

These four configurations of high PAD can be further refined into two models: the output-driven model (configuration H_1_) and the input-output linkage model (configurations H_2_, H_3_, and H_4_). Comparing configurations H_2_ and H_3_, we find that strong student involvement can substitute for strong social practice in the context of strong scientific research ability and a lack of leadership. Namely, there is a potential substitution relationship between configurations H_2_ and H_3_. Scientific research ability is a core condition in three out of four paths. Therefore, scientific research ability remains essential for improving the efficacy and development efficiency of postgraduate attributes.

### 5.2. Configuration path of low PAD

Configuration L_1_ (∼SR*∼SI*∼SP) shows that in cases featuring a lack of scientific research ability and no participation in extracurricular activities, the development efficiency of postgraduate attributes is not high. Configuration L_1_ exhibits a consistency of 0.958 and a raw coverage of 0.632, showing that this path can explain approximately 63.2% of cases. Configuration L_2_ (∼SR*∼SP*SL) shows that in cases of a lack of scientific research ability and no social practice, even if the student leadership role is fulfilled, the development efficiency of postgraduate attributes is not high. Configuration L_2_ exhibits a consistency of 0.965 and a raw coverage of 0.138, indicating that this path can explain approximately 13.8% of cases. A total of nine example cases represent this configuration. Configuration L_3_ (∼SR*∼SI*SL) shows that in cases of a lack of scientific research ability and no engagement in extracurricular activities, even if the student leadership role is fulfilled, the development efficiency of the postgraduate attribute is not high. Configuration L_3_ exhibits a consistency of 0.870 and a raw coverage of 0.142, indicating that this path can explain approximately 14.2% of cases. Configuration L_4_ (∼SR*∼MC*SL) shows that in cases of a lack of scientific research ability and no outstanding achievements in moral education, even if the student leadership role is fulfilled, the development efficiency of the postgraduate attribute is not high. Configuration L_4_ exhibits a consistency of 0.943 and a raw coverage of 0.158, showing that this path can explain approximately 15.8% of cases.

Comparing the coverages of the four configurations for low PAD discussed above, we find that configuration L_1_ exhibits the highest coverage, explaining 63.2% of cases. That is, more than half of cases emerge because path L_1_ inhibits the development efficiency of postgraduate attributes. Similarly, comparing configurations L_2_ and L_3_, we find that weak social practice and weak student involvement can be substituted for each other in a context featuring weak scientific research ability and strong student leadership. Namely, there is a potential substitution relationship between configurations H_2_ and H_3_. In addition, it is worth noting that the lack of scientific research ability is a core condition in all four paths leading to the low development efficiency of postgraduate attributes, which is consistent with Section 4.3 Necessity Analyses.

## 6. Conclusion

### 6.1. Findings

In this paper, a theoretical framework of input, environment, and output is established to evaluate the influence of extracurricular education with Chinese characteristics on postgraduate attributes via involvement, leadership, social practice, moral psychology character and research ability. To gauge the developmental efficiency of this education, we employ DEA- and fsQCA-based methods to examine various causal conditions that can enhance or obstruct postgraduate attribute development. The main findings of the study are summarized as follows: Firstly, extracurricular education with Chinese characteristics is somewhat effective in promoting postgraduate attribute development, but this efficacy is relatively modest (efficiency mean = 62.15%; 50.6% of sample attained strong DEA-efficiency), and requires improvement. Secondly, our results show that four configurations consistently result in high developmental efficiency of postgraduate attributes. Students participating in extracurricular education in an environment characterized by excellent research achievement and moral psychology attributes showed the highest developmental efficiency; while academic achievement or moral award that are insufficiently outstanding, active involvement in extracurricular activities or social practice with prosocial behavior, are also associated with high developmental efficiency. This highlights the fact that participating in extracurricular activities or social practice can provide students with valuable learning opportunities and benefit them in many ways, including developing leadership, responsibility, and social skills. Furthermore, strong student involvement and strong social practice can serve as substitutes for each other in a context featuring strong research ability and a lack of leadership, suggesting that students should be strategic in their investment of time and energy in academic research and extracurricular education. Lastly, our research reveals an asymmetric causal relationship between the high and low developmental efficiency paths, indicating that conditions affecting postgraduate attribute development exhibit multiple concurrencies (Douglas et al., 2020; Zhang and Long, 2022). All hypotheses are supported except Hypothesis 3.

### 6.2. Implications and limitations

The four configurations consistently linked to the high development efficiency of postgraduate attributes can be refined into the output-driven model (configuration H_1_) and the input-output linkage model (configurations H_2_, H_3_, and H_4_), thus indicating that postgraduate attribute development is output-oriented from the perspective of students. The input-output linkage model can explain 68.6% of cases, suggesting that graduate education should combine extracurricular education and academic research to promote the postgraduate attribute development. Classroom and laboratory learning should be linked with extracurricular activities and social practice. In addition, given the importance of scientific research ability and the substitutability between strong student involvement and strong social practice, education policy-makers should remind postgraduates to allocate time and energy efficiently.

However, there are several limitations to this study that must be acknowledged. Firstly, the sample size is limited, and the participants are drawn from a science and engineering school at a double first-class university in China. Thus, the generalizability of the conclusions is limited. Future research should explore data drawn from other countries or regions and compare our findings with those of other studies. Secondly, this study is based on students’ perspectives, and other factors such as the extra support and services provided by universities and schools may lead to different outcomes. Thus, future research should look at this issue from the perspective of schools or universities. Lastly, this study is based on cross-sectional research data collected from scholarship applications during postgraduates’ final year of education. However, postgraduate attribute development is a dynamic process, and longitudinal tracking should be adopted in future research.

## Data availability statement

The raw data supporting the conclusions of this article will be made available by the authors, without undue reservation.

## Author contributions

SL conceptualization, methodology, and writing—original draft. YH: software, data collection, and analyzing. LW supervision and writing—review and editing. EX project administration and funding acquisition. All authors contributed to the article and approved the submitted version.
